# Disease burden and social impact of pediatric chronic nonbacterial osteomyelitis from the patient and family perspective

**DOI:** 10.1186/s12969-018-0294-1

**Published:** 2018-12-14

**Authors:** Melissa Oliver, Tzielan C. Lee, Bonnie Halpern-Felsher, Elizabeth Murray, Rebecca Schwartz, Yongdong Zhao, CARRA SVARD CRMO/CNO workgroup, Melissa Oliver, Tzielan C. Lee, Elizabeth Murray, Rebecca Schwartz, Yongdong Zhao

**Affiliations:** 1 Pediatric Rheumatology, Indiana University, 699 Riley Hospital Drive, Ste 307, Indianapolis, IN 46202 USA; 2 Pediatric Rheumatology, Stanford University, Palo Alto, CA USA; 3 Pediatrics, Adolescent Medicine, Stanford University, Palo Alto, CA USA; 4 CRMO Facebook Support Group, Fort Collins, CO USA; 5 Pediatric Rheumatology, Seattle Children’s Hospital, University of Washington, Seattle, WA USA

**Keywords:** Chronic recurrent multifocal osteomyelitis, Quality of life, Patient perspective, Pediatric

## Abstract

**Background:**

Chronic nonbacterial osteomyelitis (CNO) is an autoinflammatory bone disorder that if left untreated can result in bone destruction and severe continuing pain due to persistent inflammation. The impact this chronic disease has on the daily lives of affected children and their families is not well known. The purpose of this study is to understand the disease burden and socioeconomic and psychological impact of CNO from the patients’ and families’ perspectives and identify areas of improvement for patient care and reduced disease burden based on patients’ and families’ responses.

**Methods:**

Participants were invited through a social media platform group and at clinic visits at Stanford Children’s Health. An online survey was administered to patients with a diagnosis of CNO made at < 22 years of age and/or the parent/guardian of a patient with CNO diagnosis made at < 22 years of age.

**Results:**

There was a total of 284 survey participants. The median age at CNO diagnosis was 10 years (range 2–22+). Median time from first CNO symptom to diagnosis was 2 years. Antibiotics were used in 35% of patients prior to CNO diagnosis; of these, 24% received antibiotics for greater than 6 months. Between 25 and 61% reported a negative effect of CNO on relationships, school/work performance, or finances; and 19–50% reported effects on psychosocial well-being. The majority agreed patients’ performance with daily tasks and hobbies was challenged by pain, fatigue and physical limitation related to CNO.

**Conclusions:**

Patients with CNO experienced on average a 2-year delay in diagnosis and receiving effective treatments. At least 25% reported problems with relationships, school, work, finances and well-being due to CNO. Recognition of these challenges emphasizes the need to increase awareness of this disease and address the socioeconomic stressors and mental health issues in order to provide optimal care of children with CNO.

## Background

Chronic recurrent multifocal osteomyelitis (CRMO), or the broader term chronic nonbacterial osteomyelitis (CNO), is an autoinflammatory bone disorder that typically presents as an insidious onset of bone pain with or without localized swelling, warmth and tenderness. CNO can be unifocal or multifocal at onset but the majority of children with a unifocal lesion at onset have multifocal lesions within 4 years, [1]. It commonly affects the long bones, pelvic bone, vertebrae and clavicle; however, almost any bone may be involved, so symptoms may vary, [2, 3]. If left untreated, persistent inflammation can result in bone destruction, severe continuing pain, growth disturbances, functional limitation and pathological fractures, [4–7].

There is no confirmatory diagnostic test for CNO. It is diagnosed by excluding other bone and inflammatory diseases. Due to the gradual onset, minimal physical exam findings and low sensitivity of radiographs, children with CNO may experience delays in diagnosis and access to a pediatric rheumatologist and ineffective treatments such as prolonged courses of antibiotics [2, 8–10]. Once a diagnosis of CNO is made, first line management is often nonsteroidal anti-inflammatory drugs (NSAIDs), [11, 12]. However, children with severe CNO or a recurrent multifocal course have higher rates of complications, such as pathological fractures and hyperostosis, and may require more aggressive treatment such as bisphosphonate and/or tumor necrosis factor (TNFi) inhibitors, [1, 7].

The impact this chronic disease has on the daily lives of affected children and their families may be substantial. Most studies involving children with CNO evaluated the long-term clinical outcomes from a management or disease activity perspective, [1–3, 7, 10, 13, 14]. While these studies address important concerns regarding the clinical outcomes of children with CNO, only a few have additionally addressed quality of life and psychosocial issues. In one inception cohort of 23 patients with CRMO, Huber et al. found that many patients had impairments in all domains of the quality-of-life testing, [2]. Catalano-Pons et al. evaluated educational and vocational achievements in a cohort of 40 patients with CNO and reported that a majority considered CRMO to have some global repercussion on their life and two patients reported CNO had interfered with their education, [13]. Silier et al. was the first study to highlight the difficulties of CNO from the patient perspective. In this cohort, many patients reported CNO affected their school and job attendance and had a negative influence on their family, friends, and work, [10]. However, more studies are needed to address the disease burden and the unmet need of psychosocial support for this chronic disease.

The purpose of our study was to understand the socioeconomic and psychological impact of CNO from the patients’ and families’ perspectives, with the goal of identifying areas of improvement for patient care and reduced disease burden. We surveyed patients and their families about the difficulties they encountered with the diagnosis and management of CNO and the impact on their quality of lives.

## Methods

An online survey was designed in REDCap, which is a secure, web-based application designed to support data capture for research studies, [15]. The survey consisted of 100 questions centered around three aspects: pre-CNO diagnosis management, CNO disease management and quality of life. Questions focused on epidemiological information, initial clinical presentation, physician(s) seen prior to CNO diagnosis, management before and after CNO diagnosis, diet, function and performance of daily tasks, school and work attendance, finances, peer and family relationships, fatigue, anxiety, depression, anger and sleep problems. Race and ethnicity were asked separately and were self-reported. The survey questions were developed by the study team and reviewed and refined by the parents of patients with CNO and the Childhood Arthritis and Rheumatology Research Alliance (CARRA) CRMO/CNO workgroup.

This study was approved by Stanford University Institutional Review Board (IRB Protocol # 36393) before distribution. The inclusion criteria for the survey study was patients with a diagnosis of CNO made at < 22 years of age and/or the parent or guardian of a patient with a diagnosis of CNO made at < 22 years of age. Exclusion criteria was not having a diagnosis of CNO or a parent or guardian not having a child with CNO.

Participants were recruited in two ways: 1) at their Stanford Children’s Health clinic visits, and 2) through the Facebook CRMO support group page. The Facebook group (http://www.facebook.com/groups/CRMOawareness) titled “CRMO: Chronic recurrent multifocal osteomyelitis” is an international group for patients and families dealing with CRMO. Interested participants received information regarding the study and the link to the online survey either at their clinic visits with their primary rheumatologist or through the Facebook CRMO support group webpage. The principal investigator partnered with the parent liaisons of the CARRA CRMO/CNO workgroup and the Facebook page to send reminders each month. The online survey link was available for 3 months from August 1st to October 31st, 2016. The survey was voluntary and anonymous. Informed consent was obtained online immediately prior to initiating the survey.

Descriptive statistics were performed to report the results of the survey. Results were expressed as median (ranges) for continuous variables and as frequencies (percentages) for categorical variables. Study data were collected and managed using REDCap hosted at the Stanford Center for Clinical Informatics. Statistical analysis was performed on SAS software v9.4.

## Results

A total of 350 participants initiated the survey, however, 66 responses were excluded because they did not consent (*n* = 61) or disease onset was after the age of 21 years (*n* = 5). A total of 284 survey responses were included in the final analysis, though 75 only partially completed the survey. The response rate was 26% based on 1334 members in the Facebook CRMO support group at the time of the study and 3 clinic patients.

Baseline characteristics and patient demographics are presented in Table 1. The majority of survey responses were completed by the parent or guardian (87%). Most participants were from the United States (62%) followed by: Europe (22%), Canada (7%), Australia (7%), Central/South America (1%) and other (1%). The median age of first CNO symptoms was 8 years (range 1-19y), and the median age at CNO diagnosis was 10 years (range 2-21y). The median time from the first CNO symptom to diagnosis was 2 years, with 48% of participants first seeing a pediatric rheumatologist after having 12 months of symptoms. Others reported first seeing a pediatric rheumatologist at 6–12 months (20%) and less than 6 months (32%) after their first CNO symptom. The three most common physicians seen prior to the diagnosis of CNO were primary care physicians, orthopedic surgeons, and infectious disease specialists. The rheumatologist was the fourth most common physician seen and a majority (67%) of patients were diagnosed with CNO by a pediatric rheumatologist. A bone biopsy was performed in 79% of patients for the diagnosis of CNO. The most frequent comorbidity was acne (*n* = 30, 11% of patient participants). Psoriasis was reported in 10% of patients, followed by palmar-plantar pustulosis (PPP; 8%) and juvenile idiopathic arthritis (6%).

**Table 1:** Baseline Characteristics and Patient Demographics (*n* = 284)

Characteristics		n*
Median age of first CNO symptoms, year (range)	8 (1–19)	
Median age of CNO diagnosis, year (range)	10 (2–22+)	
Survey completed by parent (%)	87	246
Male (%)	34	246
Race, white/Caucasian (%)	92	245
Ethnicity, non-Hispanic (%)	89	227
Country (%)		247
North America	70	
Europe	22	
Other	8	
Time from symptom onset and 1st pediatric rheumatologist visit (%)		239
< 6 mo	32	
6–12 mo	20	
> 12 mo	48	
Physicians seen prior to CNO diagnosis (%)		284
PCP	77	
Orthopedic Surgeon	51	
Infectious Disease	31	
Biopsy performed (%)	79	213
Received antibiotics prior to CNO diagnosis (%)	35	284
Treated with antibiotics > 6 mo	24	96
Comorbid Conditions (%)		284
Acne	11	
Psoriasis	10	
IBD	9	
PPP	8	
JIA (excluding ERA)	6	
AS/ERA	3	
Uveitis	3	

PCP = primary care physician; IBD = inflammatory bowel disease; AS/ERA = Ankylosing Spondylitis/Enthesitis related arthritis; JIA = juvenile idiopathic arthritis; PPP = Palmoplantar pustulosis; mo = month

*= number of participants who responded to survey question

**Fig. 1:**
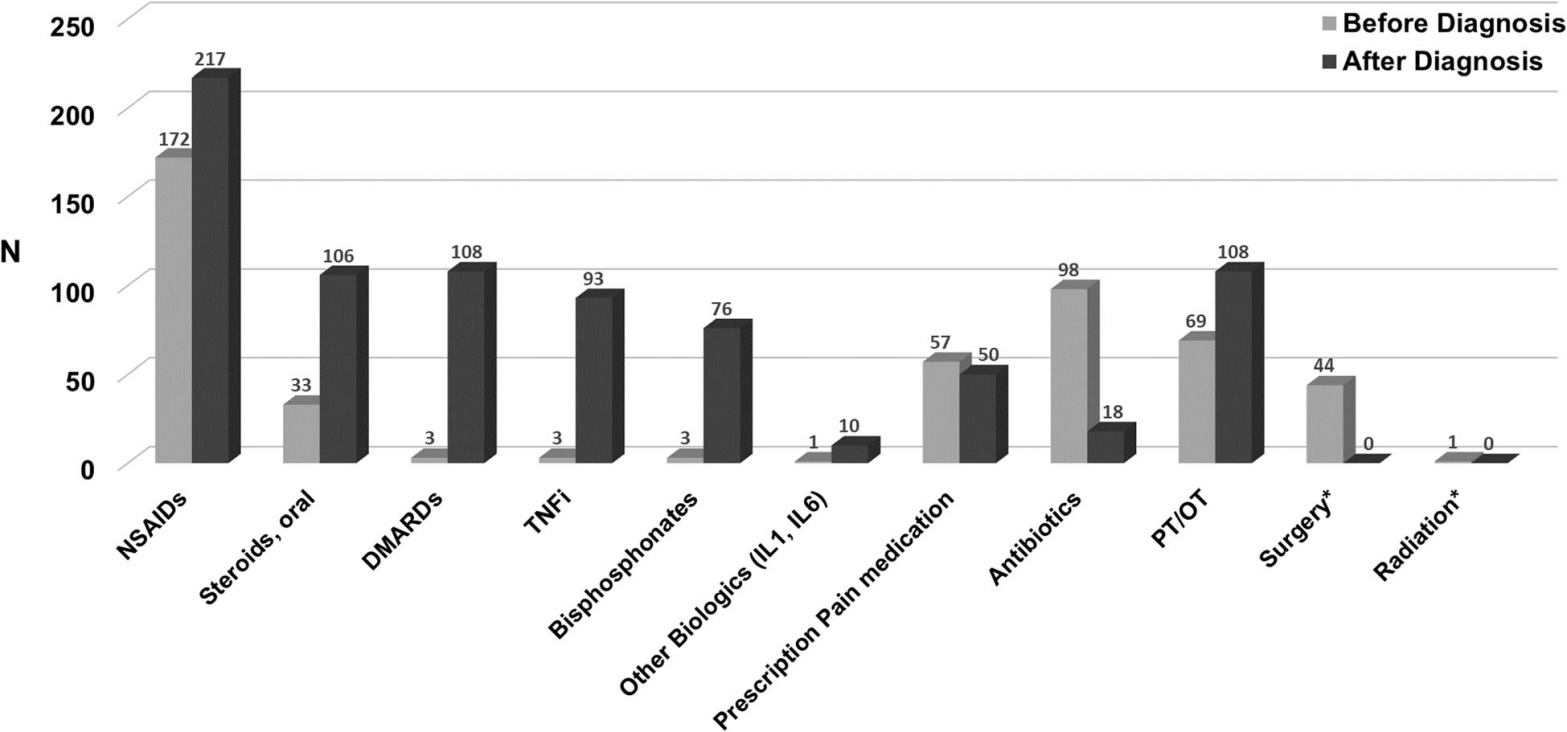
Treatments Prescribed Before and After CNO Diagnosis. Number of participants who responded to survey question for before diagnosis (*n* = 251) and after diagnosis (*n* = 248). NSAID: nonsteroidal anti-inflammatory drugs. DMARD: disease modifying anti rheumatic drug. TNFi: tumor necrosis factor inhibitor. *Surgery and radiation were not asked about after CNO diagnosis

Figure 1 displays the distribution of therapies used before and after a diagnosis of CNO. Prior to a diagnosis of CNO, NSAIDs were the most common treatment used (61%), followed by antibiotics (35%). Of those patients who were prescribed antibiotics, 24% received them for greater than 6 months duration, 12% for 3–6 months and the remaining patients received antibiotics for less than 3 months. Additional management reported prior to a diagnosis included surgery (16%) and radiation therapy (*n* = 1). After a diagnosis of CNO, NSAIDs were the first medication started in the majority of patients and the most common treatment used (88%). There was a marked increase in disease-modifying antirheumatic drugs (DMARDs), TNFi, and bisphosphonates use after a diagnosis of CNO was made compared to before. There were still 7% of patients who reported using antibiotics after the diagnosis of CNO.

Participants were asked which medication from a list of 21 choices they found to be the most helpful for the treatment of their CNO. Medications categories included steroids, antibiotics, NSAIDs, DMARDs, biologics, prescription pain medications, none and other. As shown in Table 2, in patients who also took NSAIDs at some point during their treatment, TNFi and bisphosphonate were considered to be the most helpful in 44 and 37% of participants, respectively, whereas DMARDs were considered most helpful in only 10%. It is important to note that of these patients, 65, 71 and 77% of patients were not exposed to DMARDs, TNFi or bisphosphonate, respectively. Among all the participants who took NSAIDs, 30% reported that NSAIDs were most helpful. Other medications were reported in lower numbers (prescription pain medications *n* = 5, colchicine *n* = 2, antibiotics *n* = 0 and other biologics n = 0 (i.e., IL-1 or IL-6 inhibitors)). Lastly, 27 (13%) reported that none of the medications were helpful*.* Those who reported that none of the medications were helpful also had prior use of NSAIDs (74%), DMARDs (30%), prescription pain medication (30%), TNFi (22%), bisphosphonates (22%), corticosteroids (19%) and antibiotics (15%).

**Table 2:** Medication Helpfulness in CNO/CRMO Patients with Concomitant NSAIDs Use

Medication	Reported using medication after diagnosis, n	Reported medication as most helpful*, n	% (95% CI)
TNFi	81	36	44% (33–56%)
Bisphosphonate	65	24	37% (25–50%)
NSAID	217	65	30% (24–37%)
Corticosteroids	101	17	17% (10–26%)
DMARD	100	10	10% (5–18%)

*Question formatted: “Which of the following medications has been most helpful for your CRMO/CNO?”. Total responses = 216

Other non-medicine therapeutics were commonly implemented in children with CNO. A majority (51%) reported doing physical therapy (PT) or occupational therapy (OT) for CNO-related needs. However, of those who participated in PT or OT, only 30% found it to be helpful, while 32% reported it as somewhat helpful and 38% as not helpful. Open-ended responses for why PT or OT was only partially or not helpful were: it caused more pain, there was no improvement in the pain and the physical therapist did not understand the disease. Dietary modifications for the management of CNO was also tried (35%), with gluten-free being the most common diet.

Imaging was frequently performed for the management of CNO. Almost all patients had an X-ray (91%). A CT scan was performed in 55% of patients and a bone scan in 62%. MRI was performed in 88% and whole-body MRI (wbMRI) was done for 57% of patients. However, 26.7% reported difficulty with obtaining MRI imaging. Common reasons were the availability of MRI and insurance denials.

**Fig. 2:**
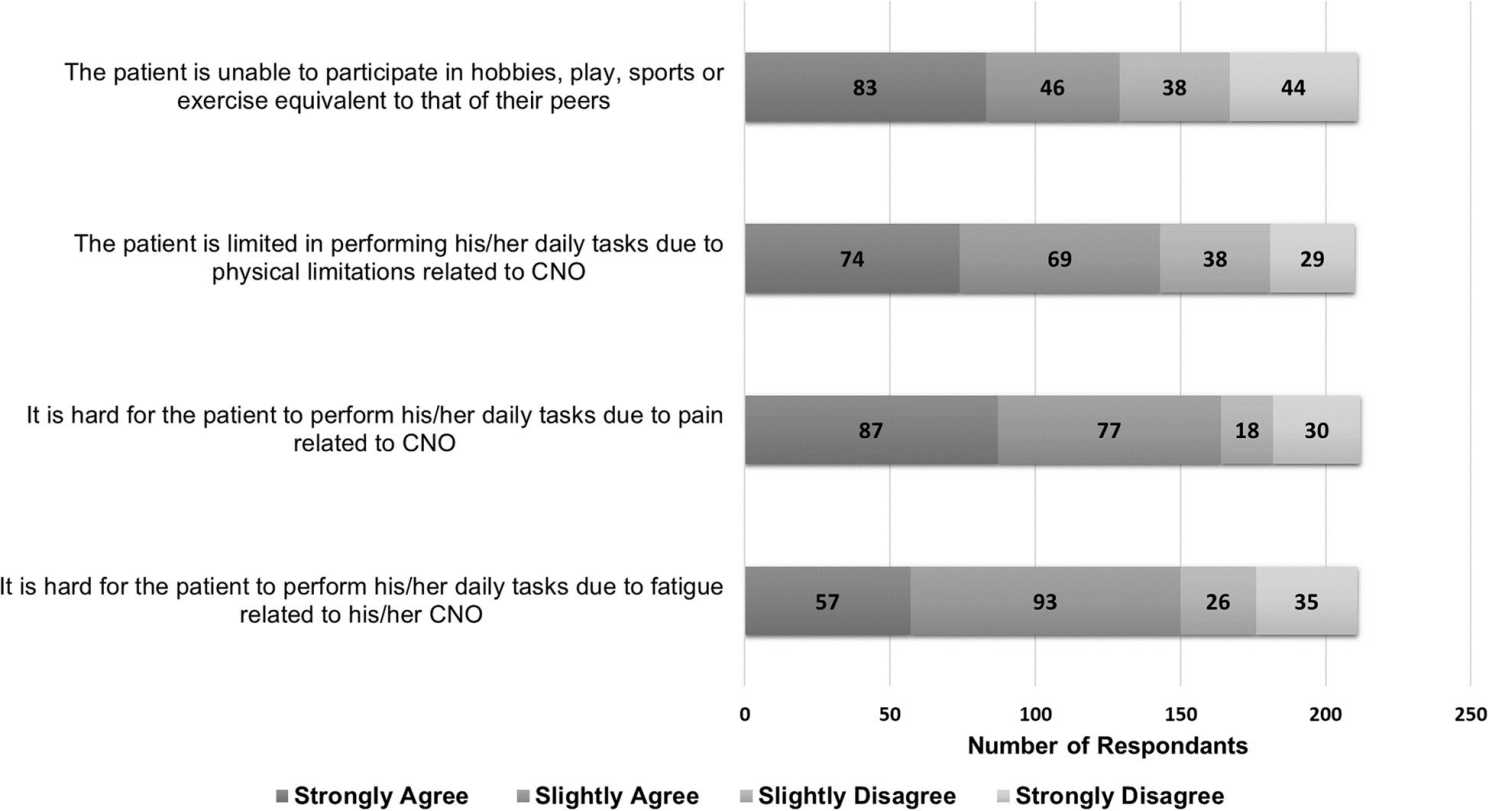
CNO Impact on Performance and Function

A majority of patients (61%) reported CNO negatively affected their participation in activities, and CNO affected their school or work attendance (53%) as shown in Table 3. Other areas that were negatively affected included school performance (36%), parent’s job attendance or performance (34%) and finances (36%). Only 36% of patients had formal school accommodations in place, such as a 504 plan. The use of assisted devices at some time during their disease course was reported by 42% of patients. Responses to the patient’s perception of how CNO affects their physical function and performance are displayed in Fig. 2. The majority agreed that the patients’ function and performance were challenged by pain, fatigue and physical limitation related to CNO and that they were unable to keep up with their peers. Of those who agreed, a significant portion had strongly agreed they were unable to perform their daily tasks due to pain (41%), physical limitations (36%) and fatigue related to CNO (28%).

**Table 3:** Self-reported Socioeconomic and Psychological Factors Affected by CNO (n = 284)

Factors	*n* (%)
Negative effect on:	
Participation in extracurricular activities	174 (61)
Patient’s school/work attendance	151 (53)
Patient’s school performance	132 (47)
Finances	101 (36)
Parent’s job attendance/performance	96 (34)
Relationship with peers	86 (30)
Relationship with parents	82 (29)
Parent’s ability to perform daily tasks	76 (27)
Relationship with siblings	71 (25)
Fatigue	143 (50)
Problems with sleep	106 (37)
Anxiety	100 (35)
General feelings of unwell	99 (35)
Depression	91 (32)
Problems with anger management	54 (19)

Additionally, in Table 3, fatigue was seen in half of the patients with CNO. Between 25 and 30% reported a negative effect on their relationships with their parents, siblings and peers. A negative effect on the patient’s psychosocial well-being was found, with 35, 32 and 19% of participants self-reporting anxiety, depression and issues with anger management, respectively.

## Discussion

Our study demonstrates the significant burden that CNO can have on the lives of patients and their families from their perspectives. Overall, patients experienced a median of a 2-year delay in diagnosis of CNO, which is similar to previously reported studies, [1, 13]. The delay may be due to a lack of awareness of CNO worldwide. Most patients will see their primary care physicians, orthopedic surgeons or infectious disease specialists prior to seeing a pediatric rheumatologist. Targeting these physicians with updated information on CNO through collaboration with pediatric rheumatologists will increase awareness and lead to earlier referrals, diagnosis and quicker initiation of appropriate treatment. Roderick et al. demonstrated that a letter sent to orthopedic surgeons led to an increase in referrals of patients with CNO to pediatric rheumatology, [8]. CNO’s insidious disease onset and the approach of diagnosis by exclusion may also contribute to the delayed diagnosis. The relative timing of the first MRI from the first symptom was not obtainable from our survey. Thus, it remains unknown whether a delayed MRI may contribute to the delayed final diagnosis.

More than one quarter reported difficulties with obtaining MRI imaging. MRI is superior to X-ray and bone scintigraphy for identifying and monitoring active CNO lesions, [16]. X-rays can be normal if done early in the disease course. Bone scintigraphy is less sensitive for determining active lesions in the metaphysis or epiphysis of the long bones in growing children. Furthermore, about 30% of patients with CNO may have asymptomatic lesions that are only detected by wbMRI, [8]. Having the advantages of no radiation and high sensitivity is why MRI, especially wbMRI, is now considered the gold standard for disease monitoring in CNO, [12, 14, 17]. More than half of the patients in our study received a wbMRI, suggesting the increasing recognition of this imaging tool in the appropriate management of CNO.

Consistent with current recommendation and practice, the most common treatment reported prior to and after CNO diagnosis was NSAIDs, [11, 18]. Antibiotic use was the second most common treatment prior to the final diagnosis, likely attributed to the difficulty in distinguishing infectious osteomyelitis from early noninfectious osteomyelitis. In our study, about one-third of patients received antibiotics prior to the diagnosis of CNO. However, what was most surprising was that one quarter used them for greater than 6 months. Prolonged antibiotic treatment has not been proven to be beneficial in patients with CNO, [3, 4, 19–21]. A consultation with a pediatric rheumatologist in these cases may reduce the duration of ineffective antibiotic treatment and can help make a timely diagnosis to begin effective interventions. After the diagnosis of CNO, the reported use of antibiotics dramatically decreased. The use of medications more appropriate for the treatment of CNO and consistent with the current clinical practices of pediatric rheumatologists increased, specifically the use of DMARDs, TNFi, bisphosphonates and steroids, [11, 12].

NSAIDs are typically the first choice for the initial treatment and are an effective therapy for CNO, [3, 7, 14]. While the majority used NSAIDs after a diagnosis of CNO, most patients did not find them to be the most helpful therapy. An even lower number of patients on DMARDs thought they were the most helpful medications, which are typically second line management. Instead, the most helpful therapies reported by patients and families for CNO management were TNFi, followed by bisphosphonates. Patients with multifocal disease or recurrences usually require more intensive therapies, but our study was not designed to differentiate disease severity among the participants.

Socioeconomic and psychological stressors related to CNO were major concerns for both the patient and the parent. Both reported interference with school and work. Overall performance and function were commonly challenged by pain, fatigue and physical limitation related to CNO. Relationships with family and peers were negatively affected. These findings are similar to the resulted by Silier et al., [18], and demonstrate an unmet need for psychosocial and socioeconomic support for the patients and families of this chronic disease. Additional support is needed for patients and their families to help cope with socioeconomic stressors and mental health matters and to establish school accommodations, such as a 504 plan, which is an education program in the United States that addresses needed accommodations for children with chronic disease to help prevent them from falling behind academically.

Our study is not without limitations. We recognized a possible sampling bias and participation bias with the online survey format and invitations through social media to collect data. However, we were able to reach a larger sample quickly, easily and on an international level through these methods, which we could not have done otherwise for this rare disease. This survey is the largest sampling of international CNO patients and their families to date. The data are self-reported and thus the accuracy of the information might be affected, particularly with the questions surrounding mental health. The majority of the survey was filled out by the parents, which may not reflect the psychosocial aspect of their children precisely. Treatment responses were also self-reported, which lack verification. While a majority had self-reported being diagnosed with CNO by a pediatric rheumatologist, we were unable to confirm the diagnosis by a health professional. Lastly, the survey was not designed to prevent participants from taking the survey more than once and a single participant could have filled out the survey multiple times.

## Conclusions

Our results emphasize the importance of understanding the challenges faced by patients and families with CNO and the need for raising awareness for this rare disease in order to improve patient care and reduce disease burden. Patients with CNO experienced on average a 2-year delay in diagnosis and receiving effective treatments. At least 25% reported problems with relationships, school, work, finances and well-being due to CNO. Our next steps should focus on educating the medical community about CNO to facilitate earlier referrals to rheumatologists for appropriate initial management, assistance with obtaining MRI imaging, guidance on effective treatments and additional support for socioeconomic stressors and mental health matters.

## Abbreviations

CNO: chronic nonbacterial osteomyelitis
CRMO: chronic recurrent multifocal osteomyelitis
DMARDs: disease modifying antirheumatic drugs
MRI: magnetic resonance imaging
NSAIDs: nonsteroidal anti-inflammatory drugs
SVARD: scleroderma, vasculitis, auto-inflammatory or other rare disease
TNFi: tumor necrosis factor inhibitors
wbMRI: whole-body MRI
